# ERK: A Double-Edged Sword in Cancer. ERK-Dependent Apoptosis as a Potential Therapeutic Strategy for Cancer

**DOI:** 10.3390/cells10102509

**Published:** 2021-09-22

**Authors:** Reiko Sugiura, Ryosuke Satoh, Teruaki Takasaki

**Affiliations:** Laboratory of Molecular Pharmacogenomics, Department of Pharmaceutical Sciences, Faculty of Pharmacy, Kindai University, Osaka 577-8502, Japan; satohr@phar.kindai.ac.jp (R.S.); takasaki@phar.kindai.ac.jp (T.T.)

**Keywords:** RAS/RAF/MEK/ERK signaling, apoptosis, DUSP/MKP, cancer treatment, ACA-28

## Abstract

The RAF/MEK/ERK signaling pathway regulates diverse cellular processes as exemplified by cell proliferation, differentiation, motility, and survival. Activation of ERK1/2 generally promotes cell proliferation, and its deregulated activity is a hallmark of many cancers. Therefore, components and regulators of the ERK pathway are considered potential therapeutic targets for cancer, and inhibitors of this pathway, including some MEK and BRAF inhibitors, are already being used in the clinic. Notably, ERK1/2 kinases also have pro-apoptotic functions under certain conditions and enhanced ERK1/2 signaling can cause tumor cell death. Although the repertoire of the compounds which mediate ERK activation and apoptosis is expanding, and various anti-cancer compounds induce ERK activation while exerting their anti-proliferative effects, the mechanisms underlying ERK1/2-mediated cell death are still vague. Recent studies highlight the importance of dual-specificity phosphatases (DUSPs) in determining the pro- versus anti-apoptotic function of ERK in cancer. In this review, we will summarize the recent major findings in understanding the role of ERK in apoptosis, focusing on the major compounds mediating ERK-dependent apoptosis. Studies that further define the molecular targets of these compounds relevant to cell death will be essential to harnessing these compounds for developing effective cancer treatments.

## 1. Introduction

**Figure 1:**
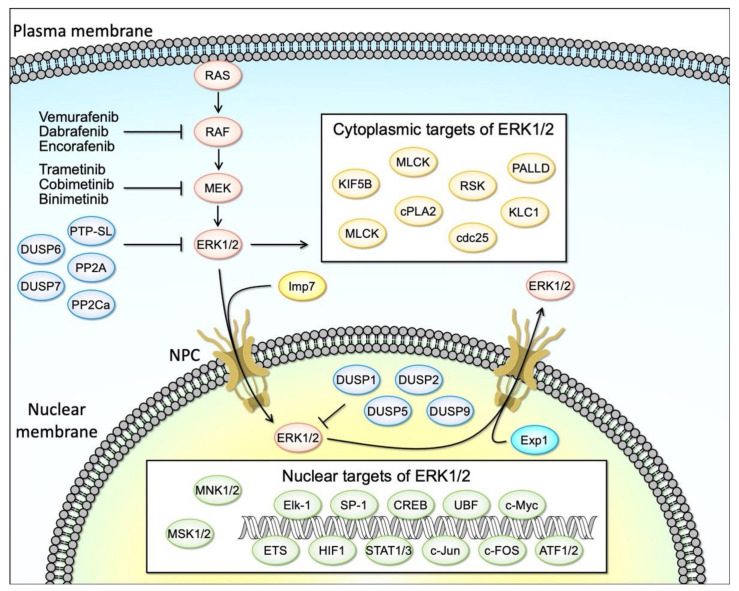
The regulation of RAF/MEK/ERK signaling pathway. The RAF/MEK/ERK signaling pathway regulates diverse cellular processes through the phosphorylation of cytoplasmic and nuclear proteins. Activated ERK1/2 translocates to the nucleus by Imp7 (Importin 7) and inactive ERK1/2 is nuclear exported by Exp1 (Exportin 1). Serine/threonine phosphatases (PP2A and PP2Ca), tyrosine phosphatases (PTP-SL), and dual-specificity phosphatases (DUSP6, 7) directly dephosphorylate ERK1/2 in the cytoplasm. Dual-specificity phosphatases (DUSP1, 2, 5, and 9) dephosphorylate ERK1/2 in the nucleus. Abnormal activation of RAF/MEK/ERK1/2 signaling is known in various cancer cells. BRAF inhibitors (vemurafenib, dabrafenib, and encorafenib), and MEK inhibitors (trametinib, cobimetinib, and binimetinib) prevent ERK1/2 activation and are currently used for cancer therapy.

The MAPK signaling pathway, comprised of RAS, RAF, MEK, and ERK is an evolutionarily conserved signaling cascade that plays a fundamental role in the control of key cellular processes, including cell survival and proliferation. Aberrant activation of RAS/RAF/MEK/ERK signaling is frequently found in various cancers, due to genetic and epigenetic alterations, rendering them attractive therapeutic targets (Figure 1) [1,2,3]. Indeed, the majority of solid tumors are explicitly characterized by their mutations in the RAS/RAF/MEK/ERK genes, wherein highly selective small molecule inhibitors of BRAF and MEK1/2 are currently used for cancer therapy (Figure 1) [4,5,6,7,8]. RAS is one of the most well-known proto-oncogenes and its gain-of-function mutations occur in approximately 30% of all human cancers [9]. RAF mutations are also frequent, particularly in melanoma [10]. Intriguingly, mutations in MEK are less identified (melanoma 3–8%, colorectal 3%) and ERK mutations have rarely been reported as drivers in human cancers despite the well-recognized importance of ERK activation in cancer malignancy [11].

Contrary to the well-established role of ERK MAPK signaling in promoting cell proliferation, survival, and tumorigenesis, growing evidence suggests that the RAF/MEK/ERK signaling can mediate pro-apoptotic signaling in vitro and in vivo [12]. In addition, various compounds with anti-cancer properties were shown to induce apoptosis in an ERK activation-dependent manner [13]. These include betulinic acid, quercetin, piperlongumine, kaempferol, and phorbol esters (DAG mimetics), and an increasing number of the compounds have been reported to exert apoptosis-inducing effects through ERK activation [14,15,16,17,18]. Furthermore, ERK activity is implicated in the mechanisms of apoptosis induced by several DNA-damaging chemotherapeutic reagents for cancer treatment as exemplified by cisplatin or etoposide (or doxorubicin) [19,20,21]. In many cases, sustained and marked ERK activation seems to be implicated in mediating apoptosis [22,23]. Impressive data have particularly highlighted the importance of the cellular threshold for active ERK1/2 levels in determining the growth arrest versus death responses mediated by ERK1/2 signaling [24]. However, despite the growing evidence of the pro-apoptotic role of ERK and the expanding repertoire of drugs capable of inducing ERK activation and apoptosis, the mechanisms underlying this Janus-faced behavior of the ERK1/2 pathway in terms of cell survival regulation remain to be fully elucidated. Recently, a chemical genetic approach using yeast genetics has identified ACA-28 and its lead compounds as ERK signaling modulators, and these compounds turned out to stimulate ERK signaling thereby inducing apoptosis specifically in ERK-active cancerous situations [25,26]. Interestingly, ACA-28 was shown to induce ERK activation and apoptosis, at least in part, via downregulation of the dual-specificity phosphatase DUSP6, a negative feedback regulator for the RAS–ERK pathway, which plays a critical role in determining the threshold of ERK MAPK activation [27].

Regarding the detailed information/literature on the anti-apoptotic role of the ERK signaling pathway in proliferation and tumorigenesis, the molecular and structural regulation of this pathway, or the possibilities of targeting this pathway for cancer therapeutics, readers are directed to excellent reviews elsewhere [7,28,29,30,31,32,33]. In this review, we will summarize recent advances regarding understanding the role of ERK in apoptosis with a special focus on the role of DUSPs in this aspect.

## 2. Overview of the RAS/RAF/MEK/ERK1/2 Signaling Pathway

The RAS/RAF/MEK/ERK pathway (hereinafter referred to as the ERK cascade) is a highly conserved signaling mechanism, which is used to connect extracellular signals from cell surface receptors to machinery that regulates multiple critical physiological processes, including growth, proliferation, differentiation, migration, and apoptosis (Figure 1) [28]. The unique feature of MAPK signaling regulation is its sequential phosphorylation cascade which, in combination with feedback loops at multiple levels, allows tight regulation of ERK signaling activity in physiological conditions.

The non-oncogenic MAPK cascade during normal growth conditions is finely regulated to ensure that ERK activation and de-activation meet the requirements of transcriptional and translational events for cell growth. ERK is activated by various extracellular stimuli such as growth factors, cytokines, hormones, and heat or oxidative stresses through receptor tyrosine kinases (RTKs) or EGFRs [34,35,36,37,38]. These receptors then transmit activating signals to the small GTPase RAS, which acts as a binary switch in growth signaling. Upon stimulation by upstream receptors, RAS switches from the GDP-bound inactive form to the GTP-bound active form. The activated RAS then goes through a conformational change in its downstream effector-binding region, leading to the binding and regulation of downstream effectors, including RAF. Activated RAF phosphorylates and activates serine/threonine-protein kinases MEK1/2, which in turn phosphorylate and activate ERK1/2 kinases [39].

It should be mentioned that there are several MAP3K isoforms (Raf-A,B,C, Mos, and MEKK1/3), and some of the alternative spliced isoforms of RAF proteins, as exemplified by a dominant-negative antagonist of A-Raf (DA Raf), antagonize their full-length counterparts and the downstream ERK pathway [40]. Interestingly, a splice isoform of human Raf1 that causes protein truncation and loss of the C-terminal kinase domain (Raf1-tr) exhibited increased nuclear localization compared with full-length Raf1. Raf1-tr was shown to have increased binding to DNA-dependent protein kinase (DNA-PK), which inhibits DNA-PK function and causes amplification of irradiation- and bleomycin-induced DNA damage. Importantly, the expression levels of Raf1-tr impacts cancer cells’ resistance/sensitivity to bleomycin-induced apoptosis. Thus, these various isoforms and spliced variants exert various cellular and pathophysiological functions, which may be mediated by antagonizing their full-length counterparts [41]. Alternative spliced isoforms of MEK (ERK1b) and ERK (ERK1c) exist as well [42], extending the signaling specificity of the cascade even further.

Activated ERK1/2 kinases phosphorylate numerous substrates which regulate cell cycle progression, differentiation, and survival (Figure 1) [30]. These substrates include cytosolic signaling proteins, p90 ribosomal S6 kinase (RSK) and MAPK-interacting serine/threonine kinase (MNK), as well as transcription factors, such as ETS domain-containing protein Elk-1, proto-oncogenes c-FOS, c-Jun and c-Myc, and cyclic AMP-dependent transcription factor ATF2, located in the nucleus [29]. RSK (also known as MAPKAP-K1) and MSK (for mitogen- and stress-activated protein kinase) constitute a family of protein kinases that mediate signal transduction downstream of MAP kinase cascades [43]. RSK is activated by the ERK family in response to various stimuli as exemplified by growth factors. In contrast, MSK is also activated by ERK in response to such stimuli, but in addition, it can be activated by p38 family MAP kinases in response to various cellular stress stimuli and pro-inflammatory cytokines/factors. In mammals, four RSK genes (RSK1-RSK4) and two MSK genes (MSK1 and MSK2) have been identified. These enzymes were among the first substrates of ERK to be discovered and have proven to be multifunctional mediators of ERK signal transduction. While the RSK isoforms promote cell survival through the inactivation of several apoptotic effectors, RSK1 and RSK2 were also shown to phosphorylate and inhibit upstream signaling components, including SOS1 and GAB2 [28]. Transcription factors targeted by the RAS–ERK pathway regulate gene expression involved in cellular proliferation, differentiation, and cell cycle progression (e.g., Cyclin D) [28,44,45]. Multiple distinct substrates of ERK1/2 have been identified to date in different subcellular compartments including the cytosol, the nucleus, or the endomembranes [46].

Positive and negative feedback plays a critical role in the maintenance of ERK activation balance and cellular homeostasis. The activity of the ERK MAPK pathway is tightly regulated by the competing actions of upstream kinases and inhibitory phosphatases as well as by the scaffolding proteins [30]. In mammals, scaffold proteins identified thus far include KSR1/2, MORG1, IQGAP1/3, β-arrestins, and LAMTOR3 [28]. Scaffold proteins fine-tune ERK signals and provide signal fidelity by isolating the ERK signaling complex from external interferences. Especially, scaffold proteins play a central role as spatial regulators of ERKs signals [47], by dictating ERK localization in the cell membrane and endomembranes via interactions with ERK. Notably, in addition to scaffold proteins, ERK signaling regulators also impact the spatio-temporal dynamics of the ERK signaling activity. For example, MEK not only activates ERK but also anchors ERK in the cytoplasm. When the signaling is in an inactive state, ERK is localized to the cytoplasm. In unstimulated cells, MEK1 binds to ERK1/2. MEK1 contains a nuclear export signal (NES) that directs its location and that of the MEK–ERK complex to the cytoplasm. The binding of MEK1 to ERK may represent a mechanism for preventing the accumulation of ERK in the nucleus where it could phosphorylate its nuclear substrates. The phosphorylation and activation of MEK1 in response to mitogenic stimuli result in dissociation of the MEK–ERK complex and the phosphorylation and activation of ERK by MEK1 [48]. The ERK cascade is inactivated by the dephosphorylation of its components by several phosphatases. These include serine/threonine phosphatases, such as PP2A and PP2Ca, tyrosine phosphatases, such as PTP-SL, as well as dual-specificity phosphatases (DUSPs), such as DUSP6, and 7 (cytosol), and DUSP1, 2, 5, and 9 (in the nucleus). DUSP5 can bind and sequester inactive ERK2 at the nucleus [49], whereas DUSP6 can retain inactive ERK2 in the cytoplasm [50], thus affecting the spatio-temporal dynamics of ERK signaling. Reciprocally, ERK activation affects DUSPs at multiple levels, through transcriptional and post-transcriptional mechanisms [51,52,53,54]. For example, DUSP6 protein expression correlates with ERK signaling activation in lung cancer cell lines, and regulation of DUSP6 is mediated at the promoter level by the ETS1 transcription factor, a well-known nuclear target of activated ERK. Another example of the negative feedback regulation of ERK signaling includes direct negative regulation of upstream kinases of the ERK signaling cascade by ERK phosphorylation. Activated cytoplasmic ERK1/2 can directly phosphorylate EGFR, RAF, and MEK, which impairs signaling as a rapid feedback response [55].

## 3. Targeting the Oncogenic Activation of the RAS/RAF/MEK/ERK Signaling for Cancer Therapy

### 3.1. Roles of ERK Signaling in Proliferation and Survival

Unlimited cell proliferation and a lack of apoptosis are important biological characteristics of tumors [56]. The ERK MAPK signaling cascade promotes proliferation and has an anti-apoptotic effect. The ERK cascade activates transcription of several proliferative signaling networks driven by FOS, Elk-1, Jun, and ETS family transcription factors, as well as Myc (Figure 1) [29]. These factors support cancer cell proliferation by promoting cell cycle entry, angiogenesis, and survival.

Apoptosis, or programmed cell death, is an essential process leading to the removal of damaged cells without affecting normal cells, following DNA damage or during development [57]. Apoptotic cell death is associated with several conserved features and culminates in the activation of cysteine–aspartic proteases (caspases), which degrade cellular components to prepare dying cells for clearance by phagocytes with minimal stress to surrounding cells and tissues [58]. Dysregulation of apoptosis has a major role in the cause of various diseases, including neurodegenerative diseases, autoimmune disorders, and cancer [59,60,61]. There are at least two broad signaling pathways that lead to apoptosis. Namely, apoptosis is initiated by either internal or external stimuli and mediated via two distinct pathways: the intrinsic pathway (mitochondria-mediated) and the extrinsic pathway [62]. The intrinsic apoptosis pathway begins when an injury occurs within the cell, which is mainly regulated by the B-cell lymphoma 2 (Bcl-2) family of intracellular proteins. Intrinsic stresses such as oncogenes, direct DNA damage, hypoxia, and survival factor deprivation, can activate the intrinsic apoptotic pathway. p53 is a sensor of cellular stress and is a critical activator of the intrinsic pathway. The extrinsic pathway begins outside a cell when conditions in the extracellular environment determine that a cell must die. Apoptotic signaling through the extrinsic pathway engaged when extracellular ligands such as TNF (tumor necrosis factor), Fas-L (Fas ligand), and TRAIL (TNF-related apoptosis-inducing ligand) are attached to the extracellular domain of the DR (transmembrane receptors), i.e., the type 1 TNF receptor (TNFR1), Fas (also called CD95/Apo-1), and TRAIL receptors.

Genetic and pharmacological evidence suggests that ERK signaling mostly provides prosurvival cues in response to serum and receptor tyrosine kinase (RTK) activation. The Bcl-2 family controls apoptosis at the mitochondria by maintaining a balance between pro-and anti-apoptotic members. The Bcl-2 family comprises anti-apoptotic (pro-survival) and pro-apoptotic members. Pro-survival members, such as Bcl-xL, are structurally similar to Bcl-2, whereas pro-apoptotic proteins Bax and Bak (Bcl-2 Antagonist Killer-1) are structurally similar to Bcl-2 and Bcl-xL and antagonize their pro-survival functions. ERK1/2 signaling regulates the activities and levels of Bcl-2 family proteins such as the pro-apoptotic protein BIM and the anti-apoptotic protein MCL-1, thereby promoting the survival of cancer cells. ERK1/2 activation can also inhibit apoptosis induced by the death receptors Fas, or TNF (extrinsic death pathway). In contrast to ERK activation, manipulation to dampen ERK1/2 signaling in response to these stimuli promotes apoptosis.

### 3.2. Roles of ERK Signaling in Tumor Development

In addition to the regulation of cellular physiological functions, such as cell proliferation, differentiation, and cell cycle, the ERK MAPK signaling is also involved in tumor formation. Elevated activation of the ERK MAPK signaling pathway has been detected in various human tumors, such as ovarian, colon, breast, thyroid, pancreatic, brain, and lung cancers [63,64,65]. Continuous activation of the ERK MAPK signaling pathway can promote the transformation of normal cells into tumors, while inhibition of the ERK MAPK signaling pathway can inhibit tumor growth in vivo [66]. Furthermore, the ERK signaling pathway plays a role in several steps of tumor development, including tumor invasion and metastasis, degradation of the tumor extracellular matrix, angiogenesis, and tumor cell migration. The phosphorylation by ERK of proteins such as myosin light chain kinase, calpain, focal adhesion kinase, and paxillin promotes cancer cell migration. Therefore, aberrant activation of ERK MAPK signaling causes de novo cell transformation and promotes tumor growth and progression.

### 3.3. Oncogenic Activation of ERK1/2 in Human Cancers

The MAP kinase cascade is probably the most important oncogenic driver of human cancers. Aberrant activation of any signaling molecule that functions upstream of ERK1/2 would culminate in the constitutive activation of these kinases, leading to tumorigenesis. Therefore, components of the ERK signaling cascade are considered attractive therapeutic targets for cancer treatment and the blockade of this signaling module by targeted inhibitors is an important anti-tumor strategy [1,5,7,33]. The ERK cascade is one of the most commonly dysregulated pathways in human cancers. KRAS is the most commonly mutated isoform in various tumor types, followed by NRAS and HRAS [67,68]. In particular, KRAS is mutated exclusively in pancreatic ductal adenocarcinoma. Oncogenic RAS mutations exhibit stable GTP-binding and constitutive activation of RAS and downstream RAF/MEK/ERK and PI3K/AKT signaling. Similarly, mutations involving BRAF, which are prevalent in malignant melanoma, exert an oncogenic effect by activating the downstream MEK/ERK MAPK pathway, resulting in uncontrolled cellular proliferation. Unlike RAS and BRAF, activating mutations in MEK are rarely found in human tumors. Intriguingly, ERK mutations are very rare in the cancer genome, although a recent paper identified ERK2 mutants as rare cancer-associated gain- and loss-of-function gene products [69].

### 3.4. Anticancer Agents Targeting the ERK Cascade

These findings propelled the development of highly selective and potent small-molecule inhibitors of kinases of the RAS-effector signaling cascades, including RAF, MEK, or ERK inhibitors for cancer therapeutics [70]. RAF monomer (BRAF (V600)) inhibitors (such as vemurafenib, dabrafenib, and encorafenib) are clinically approved for the treatment of BRAFV600 mutant melanoma (Figure 1) [33]. Given the critical role of MEK in ERK phosphorylation/activation, acting downstream of RAS and RAF, it has become an attractive drug target providing the promise of a novel therapy for RAS- and RAF-driven tumors. Indeed, two MEK inhibitors (trametinib (GSK) and cobimetinib), either as single agents or together with the RAF inhibitors (vemurafenib (Zelboraf; Genentech/Plexxikon) and dabrafenib (Tafinlar; GlaxoSmithKline)), have been approved for the treatment of patients with metastatic melanoma expressing the mutated RAF paralogue BRAF(V600E) (Figure 1). Despite intensive efforts by the investigators to search for an effective RAS inhibitor for more than three decades, oncogenic RAS mutants have been considered “undruggable” until recently due to their high affinity with GTP and the lack of a proper binding pocket for small molecule inhibitor binding [71]. It has been demonstrated that KRAS G12C could be targeted by using a covalent small molecule that docks in the switch II pocket and cross-links with Cys12 [72]. Currently, several potent covalent inhibitors against mutant-specific RAS (KRAS-G12C) are being developed and some of these molecules are in clinical trials. In contrast to the advanced development and clinical evaluation of the above-mentioned RAF and MEK inhibitors, only limited progress has been made in the ERK inhibitor development and they are still undergoing clinical trials. In contrast to RAF inhibitors, these inhibitors have a lower therapeutic index since they strongly inhibit this signaling pathway in normal cells [72,73].

Therefore, the close association of the RAS–ERK signaling activation with various clinical tumors together with the findings that the blockade of ERK signaling by specific inhibitors of RAF or MEK suppress tumor proliferation at the clinical level are consistent with the well-recognized concept that RAS–ERK pathway activation exerts anti-apoptotic functions.

## 4. Role for the ERK Cascade in Apoptosis

**Figure 2:**
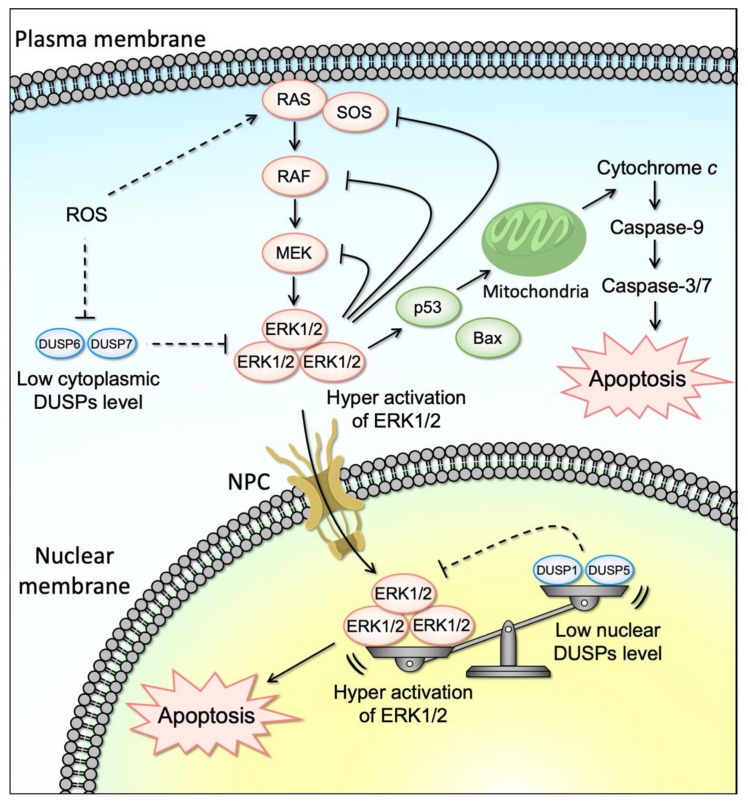
Activation of ERK1/2 upstream factors or down-regulation of DUSP6/7 by various stimuli (such as ROS) induces the activation of ERK1/2 in the cytoplasm. Activated cytoplasmic ERK1/2 induces the mitochondrial apoptosis or is translocated to the nucleus through the NPC (nuclear pore complex). Down-regulation of DUSP1/5 by the stimuli (such as ROS) also induces the activation of ERK1/2 in the nucleus. Consequently, either down-regulation of cytoplasmic or nuclear DUSPs leads to the accumulation of activated ERK1/2 in the nucleus. Sustained ERK1/2 activation above a certain threshold activates specific pro-apoptotic targets and induces apoptosis.

Notably, contrary to the well-recognized anti-apoptotic role of ERK1/2, accumulating evidence showed that ERK1/2 can be also pro-apoptotic (Figure 2). This anti-survival aspect of ERK1/2 has been observed in several physiological contexts, including neuronal cell death [74], brain injury following ischemia [75,76], development, tumor-suppressive mechanisms, and/or tumor responses to chemotherapy [77,78,79,80,81]. 

Here, we will focus on the possible mechanisms of the pro-apoptotic role of the ERK pathway in cancers by listing and analyzing the major compounds/stimuli that induce apoptosis by stimulating ERK1/2. We will also provide future perspectives on the potential and limitations of these agents as a novel cancer therapeutic approach.

### 4.1. Compounds/Stimuli That Induce Apoptosis through ERK Activation

Various agents and stimuli, which have been reported to induce apoptosis through activation of the ERK pathway are summarized (Table 1). The repertoire of the stimuli/compounds contains a broad spectrum ranging from H_2_O_2_, nitric oxide (donors), UV, γ-irradiation, phorbol esters, glutamate, cadmium, zinc, bufalin, indomethacin, to antitumor agents, including cisplatin, doxorubicin, etoposide, carboplatin, and taxol. The list of the compounds or stimuli that can induce cancer cell death by activating ERK also includes various natural compounds including resveratrol, chelerythrine, shikonin, betulinic acid, baicalein, icaritin, quercetin, piperlongumine, kaempferol, and recently identified ACA-28 and its lead derivatives. 

In most cases, the demonstration of whether the apoptosis is induced through ERK activation is experimentally investigated either by the MEK inhibitor (PD98059, U0126), ERK silencing, or the expression of the dominant-negative MEK. A paper used both MEK inhibitor and DUSP 5/6 overexpression for ERK inhibition and apoptosis cancellation [82]. Special caution should be taken when considering the results in Table 1 that use old generation inhibitors of MEK that are not as specific as the recently developed drugs were used. For example, it was shown that PD98059 can directly affect mitochondria and induce the production of reactive oxygen species [83]. Moreover, U0126 was shown to act as a direct ROS scavenger [84], implicating potential side-effects of these inhibitors on ROS signaling. In contrast, Wan et al., confirmed that U0126 did not affect ROS increase induced by 2’-dihydroxy-4,4’-dimethoxydihydrochalcone. Thus, the effect of MEK inhibitors on ROS production remains controversial. Furthermore, future work will be needed if SiRNA was used to reduce ERK activity without experiments to rule out the possible off-target effects. Some recent papers used PD184352, which is a novel, potent, and highly selective ATP non-competitive MEK1/2 inhibitor to confirm that the apoptosis is induced through ERK activation [85,86]. Future work with rigorous confirmation will further validate the properties of these compounds to induce ERK activation-dependent apoptosis.

### 4.2. Mechanisms of ERK-Induced Apoptosis

Depending on the cell types and the nature of the stimuli, ERK activation is associated with the intrinsic apoptotic pathway characterized by the release of cytochrome c from the mitochondria and activation of initiator caspase-9, or with the extrinsic apoptotic pathway, which relies on the activation of an initiator caspase-8.

In vivo studies showed that ERK1/2 kinases act upstream of caspase-3 in cisplatin-induced cell death in renal cells [87,182]. Furthermore, cisplatin-induced apoptosis required ERK activation to induce mitochondria membrane depolarization and cytochrome c release, and caspase-3 activation. Several studies suggested that ERK may act on mitochondria to induce cytochrome c release through a pro-apoptotic molecule Bax and/or p53. Cisplatin or H_2_O_2_-induced expression of Bax and p53 were decreased by the ERK pathway inhibition [183,184,185]. 

ERK also regulates the extrinsic pathway and mediates apoptosis induction. Inhibition of ERK reduced TNF-α expression, caspase-3 activation, and apoptosis in renal tissues [186]. Furthermore, inhibition of ERK activity by a MEK inhibitor U0126 abolished the increase in IL-1β mRNA induced by brain ischemia [187]. Thus, ERK may promote the expression of death ligand thereby mediating apoptosis via the extrinsic pathway.

In addition, ERK can promote cell death by suppressing the survival signaling pathways, including PI3K/AKT signaling [188,189]. Shinha et al. reported that ERK activation in response to survival factor deprivation may lead to cell death via the suppression of the survival mediated by the Akt signaling pathway [188]. Similar cross-talk between ERK and Akt signaling pathways, the two major signaling pathways relevant for survival as well as tumorigenesis was reported by various reports [190].

### 4.3. DNA Damage and p53 in the ERK Activation-Induced Apoptosis

DNA-damaging agents/stimuli are one of the largest groups that induce apoptosis via ERK activation. Chemotherapeutic agents, such as etoposide, adriamycin, platinum compounds (cisplatin), or ionizing and ultraviolet irradiation activate ERK1/2 and induced apoptosis in primary cells and various cancer cell lines [191,192]. Inhibition of ERK1/2 activity by the MEK inhibitor PD98059 attenuates apoptosis induced by DNA-damaging agents, while forced activation of ERK by the constitutively active MEK1 mutant sensitizes cells to DNA damage-induced apoptosis [191]. DNA damage is well known to activate p53 via ATM and ATR, two members of the PI3K family. Tumor protein p53 is a nuclear transcription factor that regulates the expression of a wide variety of genes involved in apoptosis, growth arrest, or senescence in response to genotoxic or cellular stresses. DNA damage induces a rapid increase in p53 protein levels and a subsequent increase in its transcriptional activity [193]. This then leads to the transcriptional activation of a number of genes whose products trigger cell-cycle arrest, DNA repair, or apoptosis, including pro-apoptotic Bcl-2 family members. The p53-mediated apoptosis pathway is one of the major apoptosis signaling pathways involving the stimulation of both the extrinsic and intrinsic pathways. p53 transcriptionally upregulates pro-apoptotic genes, Bax, Noxa, PUMA (p53-Upregulated Modulator of Apoptosis), and BID [194]. p53 has also been shown to transcriptionally repress the anti-apoptotic genes Bcl2, Bcl-xL, and Survivin [195,196]. Furthermore, p53 stimulates apoptosis via extrinsic pathway by upregulating mRNA encoding Fas and DR5 in response to DNA damage, thereby promoting cell death through caspase-3 [194]. 

Then, what is the functional connection between ERK activation and p53 in mediating DNA damage-induced apoptosis? Some studies showed that apoptosis induced by various DNA damaging agents correlates with p53 upregulation and modulation of Bcl-2 family protein in a MEK-dependent manner, suggesting that ERK activation stimulates p53 transactivation [107,114,142]. Consistently, the upregulation/accumulation of p53 by ERK activation is associated with p53 phosphorylation by ERK at Ser15 (although this Serine residue is not followed by a proline and thus does not represent a typical consensus phosphorylation site for ERK), which stabilizes p53 by inhibiting the association with Mdm2, a ubiquitin ligase for p53 [197]. In addition, phosphorylation of the Thr55 residue of p53 by ERK2 is implicated in the doxorubicin-induced p53 activation and cell death in MCF-7 breast cancer cells [198]. Furthermore, manipulating p53 activity or expression by various approaches, including p53 siRNA, a dominant-negative p53 mutant, p53 inhibitor Pifithrin-alpha, p53-deficient cells, also showed that ERK-activated apoptosis requires p53 expression [107,114,142,152,154,199]. These reports support the role of ERK in p53-mediated apoptosis in cancer cells. 

However, Tang et al., reported that etoposide-induced ERK activation takes place downstream of ATM and is independent of p53 [191], based on the findings that the MEK inhibitor failed to abolish p53 stabilization upon DNA damage and that the ERK activation in response to etoposide requires ATM but not p53. Similarly, RAS-mediated activation of ERK by cisplatin induces cell death independently of p53 in osteosarcoma and neuroblastoma cell lines, and activated ERK has been implicated in doxorubicin-mediated cell death, independent of p53 status [20,21].

Conversely, p53 may act as one of the upstream regulators of ERK activation for the induction of apoptosis in carboplatin-treated cervical cancer cells [200]. The authors reported that a requirement of ERK activation in carboplatin-induced apoptosis in SiHa and CaSki cells and that abrogation of p53 transactivation activity by a p53 inhibitor (Pifithrin-α) or dominant-negative mutant of p53 resulted in a decrease in activation of ERK in carboplatin-treated cells [200]. Another example of p53-mediated ERK activation includes ERK1/2 activation mediated by the Nutlin-3-induced mitochondrial translocation of p53. Nutlin-3, an MDM2 antagonist, induced the phosphorylation of EGFR, (MEK)1/2, and ERK1/2 in U2OS human osteosarcoma cells, which was canceled by p53 silencing [201]. 

Moreover, Heo et al. reported that DNA damage induced both the phosphorylation of p53 at Ser 15 and ERK [202]. Inhibition of p53 by a dominant-negative mutant or in p53−/− fibroblast cells abolished ERK phosphorylation and ERK inhibitor prevented p53 phosphorylation, indicating that phosphorylations of p53 and phospho-ERK are interdependent with each other. These results indicate that ATM mediates interdependent activation of p53 and ERK through the formation of a ternary complex between phopsho-p53 and phospho-ERK in response to DNA damage to cause growth arrest. Thus, ERK’s role in phosphorylating p53 in response to DNA-damaging drugs seems to be dependent on the cell line being treated.

Collectively, ERK activation plays an important role in DNA damage-induced apoptosis dependently or independently of p53. 

### 4.4. ROS and ERK Activation-Induced Cell Death

Another important signature shared by many stimuli/compounds capable of inducing ERK-mediated apoptosis is their property of simulating ROS. These include chemical oxidants such as H_2_O_2_, and nitric oxide donors, or heat, cisplatin, etoposide, doxorubicin, indomethacin, cearoin, icaritin, triptolide, and piperlongumine (Table 1). Especially, a close association between DNA damage-inducing stimuli/compounds and ROS production was reported [203]. In most cases, the demonstration of the implication of ROS in the mechanism of ERK-induced apoptosis is experimentally performed with the use of different ROS inhibitors [17,188,204,205,206,207,208,209,210].

For example, triptolide-treatment induced ERK activation in a dose- and time-dependent manner in MDA-MB-231 breast cancer cells [37]. ERK activation was crucial in mediating triptolide-induced caspase-dependent apoptosis. Triptolide-induced ERK activation modulated the expression of the Bcl-2 protein family member Bax but was not involved in the downregulation of Bcl-xL expression. ROS act upstream of ERK activation, as the MEK inhibitor U0126 did not inhibit the generation of ROS induced by triptolide treatment. Sensitization of anti-tumor agents by a compound associated with ROS-mediated ERK activation was also reported [211]. Curcumin potentiates antitumor activity of cisplatin in bladder cancer cell lines via ROS-mediated activation of ERK1/2 [114]. Importantly, NAC (ROS scavenger) and U0126 (MEK inhibitor) inhibited upregulation of p53 and apoptosis as well as downregulation of survival proteins induced by co-treatment with curcumin plus cisplatin. Thus, co-treatment with curcumin and cisplatin synergistically induced apoptosis through ROS-mediated activation of ERK1/2 in bladder cancer. 

It has long been recognized that an increase of ROS can induce or mediate the activation of the MAPK pathways, including ERK MAPK [212], and various cellular stimuli that induce ROS production can activate MAPK pathways in multiple cell types [213]. Although the precise mechanism(s) for the ROS-mediated ERK activation remains unknown, ROS can activate ERK signaling at least in two ways. Namely, activation of EGFRs, and deactivation of MAPK phosphatases (MKPs or DUSPs). ROS can specifically and reversibly react with proteins, altering their activity, localization, and half-life [214]. It is most likely that the signaling molecule action of ROS may depend upon their ability to react with the cysteine residues of a certain group of target proteins. ROS can rapidly oxidize the highly reactive thiol groups to form a disulfide bond.

Indeed, many growth factors such as EGFRs or PDGFRs, as well as MKPs/DUSPs, have cysteine-rich motifs, and they can be targets of ROS. EGFRs lie upstream of ERK and are most commonly activated through ligand-induced dimerization or oligomerization and are generally involved in the regulation of cell proliferation, survival, migration, and differentiation [215]. Interestingly, ROS have been shown to activate EGFRs even in the absence of its ligand [216], which is referred to as “receptor transactivation”. Moreover, it has been reported that oxidation of the upstream activators of ERK MAPK, including RAS, RAF, or PKC can be activated by ROS [207,208,217,218,219,220,221]. Thus, ROS can activate ERK signaling via oxidation/activation of the upstream regulators of ERK MAPK. 

By contrast, the function of MKPs/DUSPs is to dephosphorylate and, therefore, inactivate ERKs, p38 MAPKs, and JNKs. Hydrogen peroxide activates MAPK pathways, which leads to the induction of MKP-1 expression, and MKP-1 expression correlates with the inactivation of MAPK pathways [222]. Moreover, overexpression of MKP-1 renders MCF-7 cells resistant to hydrogen peroxide-induced cell death by inhibiting MAPK activation, while loss of MKP-1 by downregulation via small interfering RNA (siRNA) or deletion of the MKP-1 gene sensitizes cells to hydrogen peroxide-induced cell death through MAPK activation. This suggests that the expression levels of the MKP-1 affect MAPK activation and the sensitivity of the cells to apoptosis induced by hydrogen peroxide.

Importantly, oxidation of catalytic cysteine within the active site of DUSPs inactivates phosphatase activities of DUSPs as well as various tyrosine phosphatases [223,224]. In addition, intracellular ROS accumulation activates ERK, which triggers proteasomal degradation of DUSPs. Indeed, ROS have been shown to inhibit phosphatase activities of ERK-directed phosphatases, DUSP1 and DUSP6, by oxidation of their catalytic Cys residues. Furthermore, intracellular ROS accumulation such as hydrogen peroxide causes DUSP6 phosphorylation on Ser159 and Ser197 residues, leading to ubiquitination and degradation of DUSP6 in ovarian cancer cells. This oxidation and/or degradation-mediated inactivation/downregulation of DUSPs might contribute to the sustained ERK signaling activity, a hallmark of ERK-dependent apoptosis, which will be described later. 

### 4.5. DUSP and Negative Regulators of the ERK Cascade in the ERK Activation-Induced Cell Death

The importance of DUSP regulation in the ERK activation-mediated cell death was reported by Sugiura’s lab following their discovery of a novel ERK signaling modulator ACA-28 and its lead compound ACAGT-007. These compounds exhibited the property of inducing ERK-dependent apoptosis in human melanoma cells [25,26]. ACA-28 was isolated through a chemical genetic approach to search for compounds that can modulate ERK MAPK signaling using the fission yeast model system based on the counteractive genetic interaction between calcineurin and MAPK Pmk1 in *S. pombe* [26,225]. The unique feature of ACA-28 was to induce ERK-dependent apoptosis specifically in ERK-active human melanoma cell lines, but not in normal human melanocyte (NHEM). Similarly, using HER2-overexpressing A4-15 cells as a model system to recapitulate the cancerous situation with aberrantly active ERK, ACA-28 was shown to induce ERK-dependent apoptosis specifically in HER2-overexpressing cells, but not in the parental NIH/3T3 cells. Moreover, the IC_50_ of ACA28 against melanoma cell lines (SK-MEL-28) was much superior to that against normal cells (10.4 μM vs 5.2 μM, respectively). These results suggest that ACA-28 preferentially kills melanoma cells, and the mechanisms of the selective toxicity depend on the ERK activation status of each cell line. The analysis of DUSP6, a major ERK phosphatase in the cytosol, based on the assumption that DUSP6 regulation may be a key event to determine the ERK activation status, revealed that DUSP6 expression levels correlate with ERK activation status [27]. Namely, DUSP6 was overexpressed in HER2-overexpressing ERK-active cells as compared with NIH/3T3 cells. Furthermore, the MEK inhibitor U0126 abolished ERK activation, which led to DUSP6 downregulation. Notably, ACA-28 was shown to downregulate DUSP6 protein, at least in part, via the proteasome, and induced apoptosis in A4-15 cells by stimulating ERK phosphorylation. Consistently, DUSP6 silencing specifically induced apoptosis in ERK-active HER2 overexpressing cells, but not in NIH/3T3 cells. Thus, ACA-28 induced DUSP6 downregulation in cancer cells with highly active ERK, thereby inducing ERK hyperphosphorylation and cell death. Our study, together with the following papers showed that despite their predictive role as tumor suppressors, the ERK-inhibitory MKPs/DUSPs can be linked to tumor progression, in other words, ERK-active cancer cells are addicted to DUSPs for growth. Thus, controlling the DUSP expression can be a novel measure for cancer therapy. Consistent with the concept that DUSPs can be tumor-promoting, the MKP/DUSP inhibitor NSC 95397 reduced cell viability and anchorage-independent growth of colon cancer cell lines through inhibition of proliferation and apoptosis induction by regulating cell-cycle-related proteins and caspases [103]. Further, by using the MEK inhibitor U0126, the authors provided mechanistic evidence that the antineoplastic effects of NSC 95397 were achieved by inhibiting MKP/DUSP activity followed by ERK1/2 phosphorylation. Thus, MKP/DUSP inhibition by NSC 95397 might serve as an effective therapeutic intervention for colon cancer through regulating MKP/DUSP and ERK1/2 pathways. 

Intriguingly, Unni et al. showed that synthetic lethality induced by co-expression of mutant KRAS and EGFR is mediated through hyperactivation of ERK in lung adenocarcinoma cells, implying that tumors with mutant oncogenes in the RAS pathway must retain the ERK activity to avoid toxicities that hamper tumor growth [226,227]. DUSP6 was upregulated in EGFR- or KRAS-mutant lung adenocarcinoma cells with high ERK phosphorylation levels, which enables tumor cell growth by suppressing hyperactive ERK. Consistently, knockdown or inhibition of DUSP6 elevated phosphorylated ERK and reduced the viability of lung adenocarcinoma cells with either KRAS or EGFR oncogenic mutations, indicating that cancer cells with ERK hyperactivation with oncogene activation are addicted to DUSP high-expression for their viability. 

An additional example of the negative regulators of the ERK cascade in the ERK-dependent apoptosis includes Spry proteins, a family of endogenous proteins that negatively regulate the ERK signaling pathway. Intriguingly, similar to DUSP proteins, some cancer cells, including glioblastoma, are dependent on Spry proteins for their growth by adaptation, which makes Spry proteins play an onco-promoting role contrary to their original role as a negative regulator of the EGFR-activated ERK signaling pathway. The authors concluded that an antitumoral effect of SPRY2 inhibition is based on excessive activation of ERK signaling and DNA damage response, resulting in reduced cell proliferation and increased cytotoxicity, proposing SPRY2 as a promising pharmacological target in glioblastoma patients [228]. Altogether, compounds targeting negative regulators of the ERK cascade, such as DUSP6 and SPRY proteins, might offer a treatment strategy for certain cancers by inducing the toxic effects of RAS-mediated hyperactive ERK signaling.

### 4.6. Kinetics and Distribution of Phosphorylated ERK and Cell Death

In general, direct activation of the ERK cascade is always transient, peaking at 5–10 min after stimulation and reducing back to basal level 30–90 min thereafter. The second and third waves of activation may appear at later time points, due to autocrine loops or other ERK-required processes, such as mitosis [229]. Several compounds listed in Table 1, including H. pylori secreted protein HP1286, Etoposide, Qizhen capsule, Perfluorooctane sulfonate, Perfluorohexanesulfonate, and Cypermethrin induced ERK activation at an early stage, such as 5–15 min upon compound treatment, and U0126 abolished ERK activation and canceled apoptosis. However, various agents implicated in ERK-dependent apoptosis have also been reported to induce sustained ERK activation (Table 1). These include kaempferol, Cadmium, and Pemetrexed [17,117,123,135]. For example, Tong et al. reported that Icaritin causes sustained ERK1/2 activation and induces apoptosis in human endometrial cancer cells [23]. Icaritin treatment induced the expression of pro-apoptotic protein Bax with a concomitant decrease of Bcl-2 expression. Furthermore, icaritin induced sustained phosphorylation of ERK1/2 in Hec1A cells, and a MEK inhibitor U0126 blocked the ERK1/2 activation by icaritin and abolished the icaritin-induced growth inhibition and apoptosis. This type of long-term effects can arise from the indirect effect of other signaling pathways on the ERK activation, including the ROS-mediated oxidation and inactivation of DUSPs and other tyrosine phosphatases PP1/2A as described above (*ROS and ERK Activation-induced Cell Death*). It should be noted that many papers listed in Table 1 lack the information on the short-term effects of the compounds on ERK activation as the authors investigated ERK activation at time points wherein apoptosis was investigated (ex, 24 h). So, future work will be needed to acquire precise information in this regard.

Interestingly, transient versus sustained phosphorylation of ERK has been involved in the underlying mechanism to determine the anti-versus pro-apoptotic effects of the sex steroid estradiol. 17β-estradiol transiently induced ERK phosphorylation in osteocytic cells, whereas the estradiol-induced ERK phosphorylation in osteoclasts was sustained for at least 24 h [230]. Conversion of sustained ERK phosphorylation to a transient one abrogated the pro-apoptotic effect, whereas prolongation of ERK activation converted the anti-apoptotic effect of 17β-estradiol to a pro-apoptotic one. The authors also analyzed the effect of the nuclear export system using leptomycin (the exportin inhibitor) and further suggested that the length of time that phospho-ERKs are retained in the nucleus is responsible for the pro-versus anti-apoptotic effects of estrogen. This study suggested the intriguing possibility that not only the kinetics but also the subcellular distribution of phosphorylated ERK and its substrates in each compartment may be critical in determining the fate of the cells for anti- versus pro-apoptosis. This concept is consistent with the finding that sustained ERK activation alone, such as in models expressing constitutively active forms of upstream kinases, is not sufficient to promote cell death [231,232,233,234,235,236,237,238]. Similar sustained nuclear ERK activity was reported with apoptosis induced by tamoxifen, zinc, and doxorubicin [204,239,240].

In this regard, DUSPs again play key roles in determining both the kinetics and the distribution of phosphorylated ERK1/2. For example, DUSP6 not only serves as inactivating enzyme for ERK1/2, but it also serves as an anchor for inactive ERK in the cytosol and even participates in the transport of dephosphorylated ERKs from the nucleus to the cytosol [241]. In line with this, Kim et al. found that ROS produced during senescence of human primary fibroblasts inactivate the cytosolic ERK phosphatase DUSP6, resulting in cytoplasmic sequestration of active ERK.

Downregulation of DUSPs similar to what was observed with ACA-28 in cancer cell lines has been reported with camptothecin (CPT) in colon cancer [242]. Lee et al. reported that MKP1/DUSP1 inhibition and sustained ERK1/2 activation were implicated in CPT-induced human colon cancer cell death [242]. The authors found that CPT promoted nuclear accumulation of active ERK and prolonged ERK activity through inhibition of MKP1, implicating the function of the RAS/RAF/ERK pathway activation in cancer cell death. Among several colon cancer cell lines, CPT was shown to selectively increase the activation of ERK1/2 in HCT116 cells by downregulating MKP1 protein levels posttranscriptionally. Importantly, CPT-induced repression of MKP1 alters the nuclear-cytoplasmic location of activated (phosphorylated) ERK1/2 as evidenced by the nuclear accumulation of phospho-ERK1/2 upon CPT treatment. Thus, enhanced nuclear phospho-ERK1/2 levels were associated with MKP downregulation. These data imply that CPT-induced MKP1 protein degradation prevents the inactivation of phospho-ERK1/2 by nuclear MKP1, allowing ERK1/2 activity to be sustained in the nucleus (Figure 2). 

An additional example of the role of DUSPs in the spatio-temporal ERK signaling activation and their implication in tumorigenesis was demonstrated by Kidger et al. [243]. DUSP5 both inactivates and anchors ERK in the nucleus. The authors reported that the MKP DUSP5 terminates ERK signaling in the nucleus but, surprisingly, promotes ERK activation in the cytoplasm by relieving feedback inhibition of upstream RAF and MEK kinase activation mediated by ERK phosphorylation of each kinase (Figure 2). Importantly, DUSP5 nuclear localization is required for its effect to control cytoplasmic ERK Phosphorylation. Paradoxically, DUSP5 facilitates oncogenic mutant BRAFV600E-driven cell proliferation and transformation, suggesting that DUSP5 plays an oncogenic role in BRAFV600E-driven cancers. Consistently, BRAFV600E expression combined with Dusp5 deletion causes ERK hyperactivation and proliferative arrest, thus indicating that DUSP5 can be a novel therapeutic for cancer therapy. Notably, DUSP5 deletion does not influence normal cell proliferation in primary MEFs. Furthermore, mice entirely lacking DUSP5 develop normally and display no obvious adult phenotype, suggesting that the physiological role of DUSP5 may only be fully apparent in the event of stress or pathological challenge.

In normal cells, the subcellular localization of ERK is also tightly regulated by scaffold proteins [244,245]. Consistently, in addition to DUSPs, several cytoplasmic ERK anchoring proteins, such as DAPK, PEA-15, and Bik, have been implicated in the mechanisms of ERK1/2-mediated cell death [238,246,247]. Activated ERK interacts with these cytoplasmic anchor proteins, which sequester active ERK1/2 in the cytoplasm. Inhibition of ERK1/2 nuclear localization impairs ERK1/2-mediated survival signals and augments the pro-apoptotic signals of DAPK by ERK phosphorylation of the cytoplasmic DAPK. Consistently, mouse testes deficient in PEA-15 exhibited nuclear ERK activation [248]. Cytoplasmic sequestration of active ERK by binding to PEA-15 has been reported to induce autophagy, whereas sustained cytoplasmic ERK activity induces senescence in human primary fibroblasts. Thus, anchoring proteins for ERK, including docking phosphatases can be involved in the mechanisms of ERK-dependent cell death by affecting the distribution of phosphorylated ERK1/2 either in the cytosol or the nucleus, thereby determining the cell fate for various modes of cell death.

### 4.7. The Cellular Threshold for ERK-Dependent Cell Death

In addition to the kinetics of ERK activation, Park’s lab provided evidence regarding the threshold of active ERK that determines the ERK-dependent apoptosis versus growth arrest [24]. The authors reported that ERK1/2 overexpression switches Raf-induced growth arrest responses to caspase-dependent apoptotic death responses. Upon titration of active ERK levels by the MEK1/2 inhibitor AZD6244 reverts the death responses to growth arrest responses, suggesting that a cellular threshold for active ERK1/2 levels exists and affects the cell fate between death and growth arrest. Interestingly, kinase activity is necessary for ERK1/2 to mediate death signaling. Therefore, ERK1/2 mediates death signaling depending on kinase activity and specific physical interactions.

Consistent with these findings, our lab reported the unique feature of ACA-28 to induce apoptosis preferentially in ERK-active cancer cells, including melanoma cell lines (SK-MEL-28, SK-MEL-2, and MeWO) or HER2-overexpressing cells, but not in normal melanocyte or NIH/3T3 cells, respectively. These results suggest that cancerous situations with higher ERK phosphorylation levels were favorable for ACA-28 to induce apoptosis, consistent with a cellular threshold hypothesis. Thus, the high-ERK activation status in each cell may be a critical determinant/prerequisite for ERK-dependent cell death.

### 4.8. ERK Activation and Other Types of Cell Death

In this review, we focused on the pro-apoptotic function of the ERK signaling pathway, especially in the context of cancer cells. Recent studies highlighted the importance of the ERK cascade in various types of cell death other than apoptosis, including autophagy-dependent cell death, necroptosis, ferroptosis, pyroptosis, and parthanatos [249]. Importantly, ERK activation can also mediate other types of cell death or growth inhibitory mechanisms, including autophagy or oncogene-induced senescence [250,251,252,253]. Autophagy is an evolutionarily conserved catabolic process where cytoplasmic components and intracellular organelles are sequestered into autophagosomes and transferred to the lysosome for degradation [254]. Autophagy was initially identified as a cell survival mechanism as it constitutes an adaptive response to different kinds of stress by which the cells avoid cell death. However, in some settings, it can also contribute to a form of cell death, described as type II programmed cell death. In cancer settings, autophagy can also perform a dual role, protecting cell survival or contributing to cell death. In some tumor cells, autophagy can be a protective response enabling survival against anticancer treatments through blockade of the apoptotic pathway. Meanwhile, other tumor cells undergo autophagic cell death in response to cancer therapies [255]. 

Interestingly, some of the compounds executing ERK-dependent cell death (Table 1) simultaneously induce apoptosis and autophagy. These include cisplatin, ginsenoside Rg5 (Rg5), triptolide, morusine, scutellarin, and derrone (Table 1). For example, morusin induced a pro-apoptotic effect through the intrinsic mitochondrial apoptotic pathway and pro-autophagic effect as evidenced by the cytochrome c release and caspase-3 cleavage, as well as the increased level of LC3-II and decreased level of SQSTM1/p62, respectively [95]. ROS-mediated ERK signaling activation may constitute an underlying mechanism functionally connecting apoptosis and autophagy since the NAC treatment canceled both types of cell death associated with several compounds such as Rg5, moursine, physalin B, and derrone. For example, physalin B, a naturally occurring secosteroid, induces mitochondria-mediated ROS generation, which leads to the ubiquitin-proteasome pathway inhibition and incomplete non-canonical autophagy response [118]. Autophagosome/lysosome fusion is blocked due to the inhibition of functions of microtubules and F-actin microfilaments, leading to autophagy substrates accumulation which accelerates apoptosis. Besides, MAPK pathways are sustainedly activated and partially mediate autophagy response and apoptosis in physalin B-treated cells. Similarly, derrone, one of the major compounds of unripe fruits, induces autophagic cell death through induction of ROS and sustained ERK activation in the non-small cell lung cancer cell line [97]. In addition, activation of ERK-p53 and ERK-mediated phosphorylation of Bcl-2 is involved in autophagic cell death in several cancer cell lines, thus indicating that both ROS and p53 can also contribute to simultaneous induction of ERK-induced apoptosis and autophagic cell death.

What is the connection between autophagy and apoptosis? In the case of Rg5, suppression of apoptosis weakens Rg5-induced autophagy, while inhibition of autophagy attenuates Rg5-induced apoptosis in gastric cancer, indicating that Rg5 could simultaneously induce autophagic and apoptotic cell death in gastric cancer and that the two processes stimulate each other via activating ROS-mediated MAPK pathways [96]. Meanwhile, curcumin treatment induced autophagy via the ERK1/2 signaling pathway activation to protect chondrocytes from apoptosis [256], thus indicating the complicated interaction between apoptosis and autophagy, which can either collaborate or compete with each other.

It should be mentioned that cytosolic sequestration of phosphorylated ERK by PEA-15 can promote autophagy [257], again suggesting the importance of controlling spatial dynamics of ERK in the cell death induced by ERK activation.

## 5. Conclusions and Perspectives: Promises and Challenges for ERK-Induced Apoptosis in Cancer

ERK has largely been considered a survival signaling pathway. However, growing literature, as shown in this review, indicates that the ERK signaling pathway mediates apoptosis induced by various stimuli in different settings. Although the molecular mechanisms by which ERK mediates apoptosis remain largely elusive, several characteristics mediators of ERK activation-induced apoptosis, such as ROS and DNA damage as well as p53, are becoming clear. In addition, the importance of the spatio-temporal dynamics of ERK activation and the emerging role of the anchor proteins for ERK1/2 were highlighted. Especially, DUSPs can play critical roles in determining both the kinetics and the subcellular distribution of the ERK activation. For example, ROS can stimulate ERK signaling via activation of upstream activators and inactivation of catalytic activity of DUSPs, which leads to sustained ERK activation due to the lack of negative feedback responses elicited by DUSPs. Furthermore, compounds like ACA-28 can promote downregulation of DUSPs, which is responsible for the selective apoptosis induction in cancer cells harboring the oncogenic mutation(s) of the ERK signaling pathway. In addition to the role of ERK inactivating enzymes, DUSPs as anchor proteins for ERK can also impact the subcellular distribution of phosphorylated ERK. Thus, DUSPs can play key roles in determining ERK functions for survival or apoptosis by affecting both the kinetics/threshold (duration and magnitude) as well as the compartments of ERK activation. Sustained ERK activity in the cytoplasm might promote senescence or autophagy, whereas the sustained nuclear activity of ERK may lead to apoptosis. These distinct physiological ERK functions can be defined by ERK substrates in each compartment and the kinetics of ERK activation. For example, inhibition of ERK translocation to the nucleus hampers its access to the transcription factor substrates thereby blocking the proliferative response. Cytosolic ERK1/2, besides inhibiting the survival and proliferative signals in the nucleus, may further potentiate the catalytic activities of some pro-apoptotic proteins in the cytoplasm. Moreover, strong ERK activation above a certain threshold might activate specific pro-apoptotic targets. Similarly, sustained activation of ERK could activate additional targets that are not usually activated by transient ERK signaling. Elucidating the downstream targets of ERK1/2 responsible for the execution of distinct modes of cell death as well as the molecular mechanisms of the cellular threshold for ERK to exert apoptosis or survival will be explored in future studies. 

Inhibition of the ERK cascade (in other words, targeting the pro-survival function of ERK signaling) is the mainstream strategy for the EGFR/RAS/RAF-driven tumors. Unfortunately, however, clinical responses to the ERK cascade inhibitors are variable across patients and the efficacy of these drugs is limited and temporary. For example, despite the outstanding responses obtained with BRAF inhibitors vemurafenib and dabrafenib, the majority of patients progress within a year with a median progression-free survival of fewer than 10 months. This insufficient clinical efficacy represents tumor adaptation, largely due to the tumors’ innate or adaptive resistance to the therapies aimed at blocking the pathway activity. The most frequent response to clinical therapies is acquired resistance to drugs due to the reactivation of the ERK cascade, through mechanisms including RAF dimerization and resultant upregulation of ERK signaling. To conquer the resistance associated with ERK signaling inhibitors, several combination therapies with BRAF and MEK inhibitors, or with ERK signaling inhibitors and the PI3-kinase signaling inhibitors (PI3-kinase, mTOR, AKT) have been described. However, whether the use of such inhibitors might develop the risk of increased systemic toxicity will have to be carefully assessed.

In this regard, an emerging view that ERK1/2 can promote cell death and the strategy/compound of stimulating pro-apoptotic ERK function may reveal a new window of therapeutic opportunity for targeting cancers harboring oncogenic activations of the ERK signaling pathway. For example, sustained ERK activation status is favorable to promote cell death in several cancer cell lines [23,258,259]. Furthermore, modulating ERK activation in a given subcellular compartment might facilitate cancer cell death. It should be noted that ACA-28 preferentially kills cancer cells with high ERK activity and its lead compound ACAGT-007 showed a superior activity both in terms of efficacy and selectivity against high-ERK melanoma cells in vitro. DUSPs are highly expressed in some cancer cells with high ERK activity and this differential DUSP expression may underlie the selective apoptosis induction by ACA-28 and lead derivatives based on the cancer cells’ addiction to DUSPs. 

For the strategy to utilize ERK-induced apoptosis as an anti-cancer target, an important hurdle to overcome includes finding a means to achieve selective apoptosis induction in cancer cells with aberrant ERK activity while sparing the viability of normal cells. One way to address this issue would be a drug delivery system targeting cells expressing high-ERK phosphorylation levels by using high DUSP6 or SPRY proteins as a marker. This idea is based on the reports cited in this review, showing that inhibition/silencing of DUSP6 protein or SPRY2 protein is toxic to the oncogenic mutant lung cancer cells or glioblastoma cells, respectively, while it does not affect the viability of normal cells [227,228]. Furthermore, these negative regulators are highly expressed in cancer cells compared with normal cells. Thus, how to achieve drug delivery targeting cells expressing high DUSP6 or high SPRY proteins? For example, Ota et al. reported selective targeting of cancer cells by utilizing lysine-specific demethylase 1 (LSD1) to trigger the controlled release of anticancer drug tamoxifen in cancer cells, wherein LSD1 is highly expressed [260]. Conjugates of the LSD1 inhibitor PCPA were used as prodrugs to selectively release tamoxifen by LSD1 inhibition. Importantly, the pro-drug inhibited the growth of breast cancer cells by the simultaneous inhibition of LSD1 enzymatic activity and the estrogen receptor by tamoxifen without hampering growth in normal cells, wherein only a low amount of LSD1 is expressed with the almost undetectable release of tamoxifen. Thus, the selective release of tamoxifen in cancer cells utilizing the high expression of LSD1 in cancer cells as compared with the normal cells can be an excellent prototype for future study to achieve cancer-cell selective delivery of compounds such as ACA-28 derivatives to induce ERK-dependent apoptosis in cancer cells, but not in normal cells.

## Figures and Tables

**Table 1:** List of the compounds that induce ERK activation-dependent apoptosis.

In Vivo/Cellular Model	Stimuli Inducing Cell Death	Detected Time Points of Activated ERK	Timing of Evaluating Cell Death	Characteristics of Cell Death	Evidence Implicating MEK–ERK in Cell Death	p53- Dependent	p53 Activation	ROS Induction	Reference
RPTEC/TERT1 cells, Mouse renal proximal tubular cells	Cisplatin	72 h	48, 72 h	Caspase-3, Bcl-2, Anneexin V, Propidium iodide (PI)	Panduratin A	no information	no information	YES	[87]
Nasopharyngeal carcinoma (NPC) cell line (NPC-039 and NPC-BM)	Dehydrocrenatidine	24 h	24 h	Caspase-3, -8, -9, Bak, Bax, Bcl-xL, Bcl-2, PARP	U0126	no information	no information	NO	[88]
Human proximal tubular cell (HK-2)	Urinary proteins	2, 8 h	48 h	Annexin V	U0126	no information	no information	YES	[89]
Mouse auditory cell line (HEI-OC1)	Cisplatin	24 h	24 h	Caspase-3, TUNEL, LC3-II	U0126	no information	no information	YES	[90]
Human endometrial cancer (EC) cell (Ishikawa, HEC-1A)	Curcusone C	24 h	24 h	Caspase-3, -9	U0126	no information	no information	no information	[91]
T24	Pterostilbene, Cisplatin, Gemcitabine	48 h	48 h	LC3-II, β-galactosidase (senescence)	U0126	NO	no information	no information	[92]
Human non-small cell lung cancer (NSCLC) cell (A549, NCI-H292)	Morusin	1, 2, 4, 8, 12, 24 h	48 h	Caspase-3, PARP, LC3-II,	U0126	no information	no information	YES	[93]
Human umbilical vein endothelial cells (HUVECs)	Death-domain associated protein (DAXX) and inorganic phosphate (Pi)	1 h	24 h	Caspase-3	U0126	no information	no information	no information	[94]
LNCaP and PC-3 cells	γ-tocotrienol (GT3)	6 h	12 h	Caspase-3, -9	U0126	no information	no information	no information	[95]
Human gastric cancer cell lines (SGC-7901, BGC-823)	Ginsenoside Rg5	24 h	24 h	Caspase-3, -8, -9, PARP, LC3-II	U0126	no information	no information	YES	[96]
A549	Derrone (DR)	3, 6, 12, 24 h	24 h	Caspase-3, -8, PARP, LC3-II	U0126	YES	no information	YES	[97]
Human colorectal cancer cells (HCT116 p53+/+ and p53−/−)	6-(Methylsulfinyl)hexyl isothiocyanate (6-MSITC)	6, 12, 18, 24, 36, 48 h	48 h	Caspase-3, PARP	U0126	NO	no information	no information	[98]
HeLa	HVJ-E infection	24 h	24 h	Caspase-3, -9, LC3-II	U0126	no information	no information	no information	[99]
RSC96	Mesenchymal stem cells (MSC)-derived extracellular vesicles (EVs)	24 h	24 h	Annexin V, Bcl-2, Bax	U0126	no information	no information	no information	[100]
HL-60	Platinum complex containing a piplartine derivative cis-[PtCl(PIP-OH)(PPh3)2]PF6 (where, PIP-OH = piplartine demethylated derivative; and PPh3 = triphenylphosphine)	no infromation	24, 48 h	Annexin V, Caspase-3	U0126	no information	no information	YES	[101]
Non-small cell lung cancer (NSCLC) cells (H1975, A549)	M. tenacissima extract (MTE)	24 h	24 h	Caspase-3, PARP, LC3-II	U0126	no information	no information	no information	[102]
Non-small cell lung cancer (NSCLC) cells (PC9, H1975)	Scutellarin	48 h	24, 48 h	Annexin V, LC3-II	U0126	no information	no information	no information	[103]
Colon cancer cell lines (SW480, SW620, DLD-1)	NSC95397	6 h	24 h	Caspase-3, -7, -9, PARP	U0126	NO	no information	no information	[104]
Human breast cancer cell line (MDA-MB-231)	Triptolide	2, 4, 8, 24, 48 h	48, 72 h	Caspase-3, PARP	U0126	no information	no information	YES	[37]
Human breast cancer cell (MCF-7)	Triptolide (TPI)	24 h	24 h	Caspase-3, LC3-II	U0126	no information	no information	no information	[105]
MKN45	Alpha, 2′-dihydroxy-4,4′-dimethoxydihydrochalcone	48 h	48 h	Annexin V, LC3-II	U0126	no information	no information	YES	[106]
Non-small cell lung carcinoma (NSCLC) (A549, H226, H1299)	Artocarpin	0.5, 1, 2, 3, 4 h	6, 24 h	Caspase-3	U0126, SB202190	dependent and independent	YES	YES	[107]
Murine macrophage cell line RAW264.7	H. pylori secreted protein HP1286	15, 30, 60, 90, 120 m	24 h	Caspase-3	U0126	no information	no information	no information	[108]
HepG2	Cholix toxin (Cholix)	4, 8, 12 h	12 h	Caspase-3, -9	U0126, SB20852, SP600125	no information	no information	YES	[109]
Human ovarian cancer cell lines (HEY, A2780)	Baicalein (BA)	24 h	24 h	LC3-II, PARP	U0126, ERK siRNA	no information	no information	no information	[110]
MGC-803	Equol	12, 24, 48 h	24 h	Caspase-3, PARP, cIAP1	U0126	no information	no information	no information	[111]
A549	Derivatives of 6-cinnamamido-quinoline-4-carboxamide (CiQ)	8 h	48 h	Caspase-9, PARP, LC3-II	U0126, dominant-negative MEK1	NO	no information	no information	[112]
Human nasopharyngeal carcinoma (NPC) cell lines (HONE-1, NPC-039)	Polyphyllin G (polyphyllin VII)	3, 6, 12 h	24 h	Caspase-8, -3, -9, Bax, Bcl-xL, Bcl-2, LC3-II	U0126	no information	no information	no information	[113]
253J-Bv, T24	Co-treatment of curcumin and cisplatin	3, 6, 24 h	24 h	Caspase-3, Anneexin V	U0126	dependent and independent	YES	YES	[114]
Burkitt’s lymphoma Ramos cells	MytiLec	12, 24 h	24 h	Caspase-3, -9, Annexin V	U0126	no information	no information	no information	[115]
Human umbilical vein endothelial cells (HUVECs) (SGC7901)	farrerol	24 h	24 h	G0/G1 cell cycle arrest	U0126	no information	no information	no information	[116]
Osteoblasts (OBs)	Cadmium (Cd)	2, 3, 4, 5, 6 h	24 h	Bax, Bcl-2, Caspase-3, -9, PARP	U0126	no information	no information	Decrease in ROS generation	[117]
HCT116	Physalin B	12, 24, 36 h	24 h	Caspase-3, PARP, LC3-II	U0126	no information	no information	YES	[118]
NIH/3T3	Paraquat	0.5, 1, 6, 12, 24 h	24 h	Cytochrome c	U0126	no information	no information	YES	[119]
Breast cancer cell lines (MCF-7, BT474, MDA-MB-231, SUM1315)	D Rhamnose β-hederin (DRβ-H)	5, 30 m, 2, 6, 48 h	48 h	Annexin V, caspase-3, -8, -9	U0126	no information	no information	no information	[120]
A549	Pyocyanin (PCN)	5 m, 1, 3, 6, 12, 24 h	24 h	Caspase-3	U0126	no information	no information	YES	[121]
Mouse TM4 Sertoli cells	Nonylphenol (NP)	15, 30, 60, 180 m	24 h	Sub G1, Caspase-3, PARP, Bcl-2, Bax	U0126	no information	no information	YES	[122]
HT-29	Piperlongumine (PPLGM)	5, 10, 15, 30, 60 m	24 h	Caspase-3	U0126	no information	no information	no information	[16]
BRL 3A	Cadmium (Cd)	12 h	12 h	Annexin V, Bax, Bcl-2	U0126	no information	no information	YES	[123]
Rat liver cells	Corticotrophin-releasing hormone (CRH)	8 h	8, 24 h	Bax, Bcl-2	U0126	no information	YES	no information	[124]
MDA-MB-468	Allyl isothiocyanate (AITC)	2 h	12 h	Caspase-3, -9, Bcl-2, Cytochrome c	U0126	NO	NO	YES	[125]
Mouse embryonic stem (mES) cells	Sodium nitroprusside (SNP)	4, 8, 12, 24 h	24 h	Caspase-3, -8, -9, Annexin V	U0126	no information	no information	YES	[126]
SGC-7901	β,β-Dimethylacrylshikonin	2, 4, 8, 12, 24 h	48 h	Caspase-3, -8, -9, Bax, Bcl-xL, Bcl-2, PARP, Cytochrome c	U0126	no information	no information	no information	[127]
H9c2	Cyclosporine A (CsA)	0.25, 0.5, 1, 4, 8, 12, 24 h	4 h	Caspase-3, Bax, Bcl-2, Annexin V, TUNEL	U0126	no information	no information	no information	[128]
Breast cancer cell lines (MDA-MB-231, KPL-3C)	Olaparib	1, 12 h	12, 24 h	Annexin V	U0126	no information	no information	no information	[129]
Jurkat/NTAL(+)	Methylprednisolone	5, 30 m	24 h	Annexin V, Propidium iodide (PI)	U0126	no information	no information	no information	[130]
Jurkat	Phorbol myristate acetate (PMA)	24 h	16, 24 h	DNA fragmentation	U0126, PD98059	no information	no information	no information	[131]
Head kidney macrophage (HKM)	Arsenic	24 h	24 h	Caspase-3	U0126	no information	no information	YES	[132]
LLC-PK1	Cysteinyl leukotrienes (cysLTs) synthesis	0.5, 1, 3, 6, 12, 24 h	24 h	Caspase-3, Bax, Bcl-2, Cytochrome C	U0126	no information	no information	no information	[133]
HeLa	Phenethyl isothiocyanate (PEITC)	8, 24 h	24 h	Caspase-3, PARP	U0126	no information	no information	no information	[134]
Human lung adenocarcinoma A549	Pemetrexed	4, 8, 12, 24, 48 h	72 h	Caspase-3, TUNEL	U0126, PD98059, ERK siRNA	no information	no information	no information	[135]
Human endometrial cancer Hec1A	Icaritin	3, 6, 12, 24 h	24 h	Caspase-3, -9, PARP, Cytochrome c, TUNEL, Annexin V	U0126	no information	no information	no information	[23]
Breast cancer MDA-MB-453 and MCF7	Icaritin	5, 15, 30 m, 1, 2, 4, 8, 12, 24 h	48 h	Bcl-2, PARP, Annexin V	U0126	no information	no information	no information	[136]
Renal tubular epithelial cell (RTEC)	HIV-1 viral protein r (Vpr)	5 d	5 d	Caspase-3, -8, -9, PARP	U0126	no information	no information	no information	[137]
Spinal cord	Spinal cord ischemia/Reperfusion (I/R) injury	3 h	24 h	Caspase-3, TUNEL, c-IAP2	U0126	no information	no information	no information	[138]
HL-60, U397, SK-MEL-1	Asteriscunolide A (AS)	0.5, 1, 2, 3 h	24 h	Caspase-3, -7, -9, PARP	U0126, PD98059	no information	no information	YES	[139]
Astrocytes	Glutamate	3, 6, 9 h	24 h	Caspase-3, TUNEL	U0126, DUSP5, 6 over-expression	no information	no information	no information	[82]
Neuro-2a	4-Methyl-2,4-bis(4-hydroxyphenyl)pent-1-ene (MBP)	0.5, 1, 2, 4, 6, 8 h	24 h	Sub-G1, Caspase-3, -7, -9, -12, Bax, Bcl-2, PARP, Cytochrome c	PD98059	no information	no information	no information	[140]
Human platelets	Bisdemethoxycurcumin (BDMC)	1, 2 h	1 h	Caspase-3, -8, -9, Bax, BID, Bcl-2, Bcl-xL, Cytochrome c	PD98059	no information	no information	YES	[141]
HeLa, MCF-7	*N*-Methyl and *N*,*N*-dimethyl bis(indolyl)hydrazide-hydrazone analog derivatives	no information	24 h	Bak	PD98059	YES	YES	YES	[142]
Human ovary adenocarcinoma (SKOV3)	CRT1 (Ent-18-acetoxy-7β-hydroxy kaur-15-oxo-16-ene)	24 h	24 h	Caspase-3, -7, -9, PARP, Cytochrome c, Annexin V, Propidium iodide (PI)	PD98059	no information	no information	no information	[143]
Human pulmonary microvascular endothelial cells (HPMECs)	Cigarette smoke extract (CSE)	12 h	12 h	Caspase-3, TUNEL, Annexin V, Propidium iodide (PI)	PD98059	no information	no information	no information	[144]
Hepatocellular carcinoma (HCC) (SMMC7721, Bel7402)	Hispidulin	48 h	48 h	Caspase-3, Bax, Bcl-2, Annexin V, Propidium iodide (PI), TUNEL	PD98059	no information	no information	no information	[145]
Human osteosarcoma cells (HOS)	Honokiol (HNK)	24 h	24 h	Caspase-3, -9, Bcl-2, Bcl-xL, survivin, PARP, Annexin V, Propidium iodide (PI), TUNEL	PD98059	no information	no information	YES	[146]
Human hepatoma (Hep3B)	Desipramine	1, 2, 4, 8 h	24 h	LDH	PD98059	no information	no information	YES	[147]
Human neuroblastoma (SH-SY5Y)	Cearoin	12 h	12 h	Caspase-3, Bax, Bcl-2, PARP	PD98059	no information	no information	YES	[148]
Human Glioma (U87)	Valproic acid (VPA)	72 h	72 h	Hoechst 33342, Caspase-3, -9, Bax, Bcl-2, Cytochrome c, TUNEL, Annexin V, Propidium iodide (PI)	PD98059	no information	no information	no information	[149]
Human gastric cancer cells (AGS)	Agrimonolide (AM)	24 h	24 h	Caspase-3, -8, -9, Bax, Bcl-2, Annexin V, Propidium iodide (PI)	PD98059	no information	no information	YES	[150]
β-cell-derived cells (RIN-m5F)	Etoposide	15, 30, 60 m	24 h	SubG1, Caspase-3, -6, -7, -9, Cytochrome c, Bax, Bcl-2, Annexin V, Propidium iodide (PI)	PD98059	no information	no information	no information	[19]
HT-29	Simvastatin	24 h	48 h	Caspase-3, Bax, Bcl-2	PD98059	no information	no information	no information	[151]
Ardiac myocytes (H9c2)	Doxorubicin (DOX)	2, 6, 12, 24, 48 h	24 h, 48 h	TUNEL, Annexin V, Propidium iodide (PI)	PD98059, ERK siRNA	YES	YES	no information	[152]
RAW 264.7 cells	Cypermethrin	15, 30 m	48 h	Annexin V, Propidium iodide (PI)	PD98059	no information	YES	YES	[153]
Male Sprague–Dawley rats, Primary hippocampal neuron cells	Early brain injury (EBI) following subarachnoid hemorrhage (SAH), oxyhemoglobin (OxyHb)	6, 12, 24, 48 h	24 h	Caspase-3, -8, -9, PARP, TUNEL	PD98059	YES	YES	YES	[154]
Neuroblastoma cells (neuro-2a)	Honokiol (HNK)	48, 72 h	72 h	BrdU	PD98059	no information	no information	YES	[155]
Human extravillous cytotrophoblast (EVCT)-derived cells (HTR-8/SVneo, HPT-8)	Coplanar polychlorinated biphenyls (Co-PCBs)	24 h	24 h	Annexin V, Propidium iodide (PI)	PD98059	no information	no information	no information	[156]
Human multiple myeloma (MM) cells (U266)	Cinobufagin (CBG)	3 h	24 h	Caspase-3, Bcl-xL, Bcl-2, PARP, Survivin, Annexin V, Propidium iodide (PI)	PD98059	no information	no information	YES	[157]
Lung adenocarcinoma cells (A549)	Triptolide (TP)/Hydroxycamptothecin (HCPT)	24 h	24 h	Caspase-3, -9, Cytochrome c, Bax, Bcl-2, Annexin V, Propidium iodide (PI)	PD98059	no information	no information	no information	[158]
Human leukemia cells (HL-60)	Bromo analogue (TBr)	6, 12, 24 h	24 h	Caspase-3, -8, -9, PARP, Bax, BID, Bcl-2, Sub-G1, Annexin V, Propidium iodide (PI)	PD98059	no information	no information	no information	[159]
Human alveolar cells (A549)	Acetochlor (ACETO)	6, 12, 24, 48 h	72 h	Caspase-3, -9, PARP, Bak, Bax, BID, Bad, MCL-1, Bcl-xL, Bcl-2, Annexin V, Propidium iodide (PI)	PD98059	no information	no information	YES	[160]
PC12	Perfluorohexanesulfonate (PFHxS)	0.5, 1, 3, 6, 9, 16, 24 h	24 h	Caspase-3, Propidium iodide (PI), TUNEL	PD98059	no information	no information	YES	[161]
Prostate cancer cells (PC3)	MHY-449	24 h	24 h	Caspase-3, -8, Bax, Bcl-2, PARP, Annexin V, Propidium iodide (PI)	PD98059	NO	no information	no information	[162]
Neuro-2a	As2O3	15, 30, 60 m	24 h	Caspase-3, -6, -7, -9, -12, PARP, Bax, Bcl-2,	PD98059	no information	no information	YES	[163]
Gastric cancer cells (SGC7901, MGC803)	Interferon-α (IFN-α)/5’-Deoxy-5-fluorouracil (5’-DFUR)	24, 48 h	24 h, 48 h	Caspase-3, -9, PARP	PD98059, ERK siRNA	no information	no information	no information	[164]
Osteosarcoma cells (G292)	Capsaicin	2, 6 h	24 h	Caspase-9, Annexin-V	PD98059	no information	no information	no information	[165]
Rat chondrocyte	Mechanical stress (0.5 MPa)	24 h	24 h	Caspase-3, -9, Bax, Bcl-2, Annexin V, Propidium iodide (PI), TUNEL	PD98059	no information	no information	no information	[166]
Leukemia cells (KBM-5)	Tanshinone IIA (Tan IIA)	6, 12, 18, 24 h	24 h	Sub-G1, PARP	PD184352	no information	no information	no information	[85]
Rat hepatic stellate cell (HSC-T6)	Tanshinone IIA (Tan IIA)	6 h	24 h	Sub-G1, Caspase-3, -9, PARP, Bax, Bcl-2	PD98059	no information	no information	YES	[167]
Cerebellar granule cells (CGCs)	Perfluorooctane sulfonate (PFOS)	5, 10, 15, 30 m, 1, 3, 6 h	24 h	Hoechst 33342, Caspase-3	PD98059	no information	no information	no information	[168]
Human oral squamous cell carcinomas (YD-10B)	Fomitoside-K	4, 8, 12, 24 h	24 h	Caspase-3, -9, PARP, Bax, Bcl-2, Survivin, Propidium iodide (PI)	PD98059	no information	no information	YES	[169]
Human myeloid leukemia cells (K562)	Calactin	6, 12, 24 h	12 h, 24 h	Caspase-3, -8, Caspase-9, Bax, Bcl-2, Annexin V, Propidium iodide (PI)	PD98059	no information	no information	no information	[170]
Human gastric cancer cells (CS12)	Euphol	4, 24, 48, 72 h	48 h, 72 h	Caspase-3, Annexin V, Propidium iodide (PI)	PD98059	no information	no information	no information	[171]
Human oral squamous cell carcinoma (OSCC) cells (YD-8)	P. densiflora leaf essential oil (PLEO)	4, 8 h	8 h	Caspase-9, PARP, Bax, Bcl-2	PD98059	no information	no information	YES	[172]
Osteoblasts (Saos-2)	CdCl2	3, 4, 18 h	48 h, 72 h	APO *Percentage* dye	PD98059	no information	no information	no information	[173]
Human hepatoma (HepG2)	ZL11n	24 h	24 h	Caspase-3, Bax, Bcl-2, Annexin V, Propidium iodide (PI)	PD98059	no information	no information	no information	[174]
Amygdala region in SPS rats	Single-prolonged stress (SPS)	1 h	1 h, 7 d	Bax, Bcl-2, TUNEL	PD98059	no information	no information	no information	[175]
Human myeloid leukemia cells (K562)	Ormeloxifen (ORM)	1, 3, 6, 24 h	48 h	Caspase-3, -9, Bax, Bcl-2, Annexin V, Propidium iodide (PI), TUNEL	PD98059	no information	no information	no information	[176]
HK-2	Cyclosporine A (CyA)	1, 3, 6, 24 h	24 h	Hoechst 33258	PD98059	no information	no information	no information	[177]
PC12	Parathyroid hormone (PTH)	24, 48, 72 h	24, 48, 72, 96 h	Caspase-3, Propidium iodide (PI), DNA ladder	PD98059	no information	no information	no information	[178]
HCT116	Qizhen capsule (QZC)	15, 30, 60 m	24 h, 48 h	Caspase-3, -9, Bax, Bcl-2, PARP, Annexin V, Propidium iodide (PI)	PD98059	no information	no information	no information	[179]
Human melanoma cells (A375SM), mouse xenograft model	Shikonin	24 h	24 h	Bax, Bcl-2, PARP, Annexin V, propidium iodide (PI), TUNEL	no information	no information	no information	no information	[180]
Rat hepatocytes	Di-(2-ethylhexyl)phthalate (DEHP)	24 h	24 h	Caspase-3, PARP, Bad, Bax, Bcl-2, Bcl-xL, Annexin V, Propidium iodide (PI)	PD98059	no information	no information	YES (ROS independent)	[181]
Human leukemia cell line (K562)	Prodigiosin	48 h	24, 48 h	Caspase-3, -8, -9, Annexin V, LC-3	PD184352	no information	no information	YES	[86]
